# Commensal *Bifidobacterium* Strains Enhance the Efficacy of Neo-Epitope Based Cancer Vaccines

**DOI:** 10.3390/vaccines9111356

**Published:** 2021-11-18

**Authors:** Michele Tomasi, Mattia Dalsass, Francesco Beghini, Ilaria Zanella, Elena Caproni, Laura Fantappiè, Assunta Gagliardi, Carmela Irene, Enrico König, Luca Frattini, Giulia Masetti, Samine Jessica Isaac, Federica Armanini, Fabio Cumbo, Aitor Blanco-Míguez, Alberto Grandi, Nicola Segata, Guido Grandi

**Affiliations:** 1 Department of Cellular, Computational and Integrative Biology, University of Trento, 38123 Trento, Italy; michele.tomasi.2@unitn.it (M.T.); mattia.dalsass@unitn.it (M.D.); francesco.beghini@unitn.it (F.B.); ilaria.zanella@unitn.it (I.Z.); elena.caproni@unitn.it (E.C.); carmela.irene@unitn.it (C.I.); enrico.koenig@unitn.it (E.K.); luca83@gmail.com (L.F.); giulia.masetti@unitn.it (G.M.); samine.isaac@gmail.com (S.J.I.); federica.armanini@unitn.it (F.A.); fabio.cumbo@unitn.it (F.C.); aitor.blancomiguez@unitn.it (A.B.-M.); nicola.segata@unitn.it (N.S.); 2 Toscana Life Sciences, 53100 Siena, Italy; l.fantappie@toscanalifesciences.org (L.F.); a.gagliardi@toscanalifesciences.org (A.G.); a.grandi@toscanalifesciences.org (A.G.); 3 BiOMViS Srl, Via Fiorentina 1, 53100 Siena, Italy

**Keywords:** cancer vaccines, microbiome, immunotherapy, OMVs, personalized medicine

## Abstract

A large body of data both in animals and humans demonstrates that the gut microbiome plays a fundamental role in cancer immunity and in determining the efficacy of cancer immunotherapy. In this work, we have investigated whether and to what extent the gut microbiome can influence the antitumor activity of neo-epitope-based cancer vaccines in a BALB/c-CT26 cancer mouse model. Similarly to that observed in the C57BL/6-B16 model, *Bifidobacterium* administration per se has a beneficial effect on CT26 tumor inhibition. Furthermore, the combination of *Bifidobacterium* administration and vaccination resulted in a protection which was superior to vaccination alone and to *Bifidobacterium* administration alone, and correlated with an increase in the frequency of vaccine-specific T cells. The gut microbiome analysis by 16S rRNA gene sequencing and shotgun metagenomics showed that tumor challenge rapidly altered the microbiome population, with *Muribaculaceae* being enriched and *Lachnospiraceae* being reduced. Over time, the population of *Muribaculaceae* progressively reduced while the *Lachnospiraceae* population increased—a trend that appeared to be retarded by the oral administration of *Bifidobacterium*. Interestingly, in some *Bacteroidales*, *Prevotella* and *Muribaculacee* species we identified sequences highly homologous to immunogenic neo-epitopes of CT26 cells, supporting the possible role of “molecular mimicry” in anticancer immunity. Our data strengthen the importance of the microbiome in cancer immunity and suggests a microbiome-based strategy to potentiate neo-epitope-based cancer vaccines.

## 1. Introduction

Approximately 10^14^ bacteria belonging to thousands of species proliferate in the 200 m^2^ of human intestine, and from birth they exert a crucial role in programming innate and adaptive immune responses [1]. Unbalance and alteration of the gut microbiome can result in severe pathological immune responses and can alter therapeutic regimes. For instance, a number of studies support the notion that the remarkable increase in allergies and autoimmune diseases such as type I diabetes and lupus erythematosus observed over the last century in industrialized countries could be attributed to a reduced exposure to environmental microbes and pathogens and to excessive use of antibiotics [2]. More recently, a large amount of data in both animals and humans demonstrate that the gut microbiome also plays a fundamental role in cancer immunity and in determining the efficacy of cancer immunotherapy [3]. A recent epidemiological study has shown that antibiotic-associated dysbiosis can enhance the frequency of certain cancers, including lung, prostate and bladder cancers [3,4]. Furthermore, Sivan and coworkers showed that C57BL/6 germ-free or microbiome-depleted mice respond poorly to PD-1/PD-L1 therapy, while the antitumor activity of checkpoint inhibitors is potentiated when *Bifidobacterium* species are administered by oral gavage after tumor challenge [5]. The beneficial effect of *Bifidobacterium bifidum* on anti-PD-1 therapy was also recently demonstrated in a CT26-BALB/c mouse model [6]. Moreover, transplantation of fecal microbiome from patients responding to PD-L1 therapy, but not from non-responders, improves the efficacy of checkpoint inhibitors both in animal models and in melanoma patients [4,7,8]. Finally, retrospective analyses in human patients under PD-1/PD-L1 therapy show the deleterious effect of antibiotics administered during the mAb treatment [3].

The mechanisms through which the gut microbiome influences the antitumor activity of checkpoint inhibitors remain to be fully elucidated [3]. It has been clearly shown that some gut-associated microbial species can enhance the frequency of antitumor T cells both in circulation and at tumor site [5], even though the antigen specificity of such T cells has not been characterized yet. On the other hand, immune checkpoint inhibitors act by removing the inhibitory pathways that block antitumor T cells, and it is well documented that their efficacy is directly proportional to tumor mutational burden, which generates cancer-specific neo-epitopes recognized by CD8^+^/CD4^+^ T cells [9]. Therefore, the amplification of neo-epitope-specific T cells is likely to be the common denominator of the antitumor activity of both gut microbiome and checkpoint inhibitors, and can explain their cooperative effect.

The discovery that tumor-associated genetic mutations create neo-epitopes which become the target of antitumor T cells has revitalized the field of cancer vaccines, whose efficacy had been so far largely disappointing [10]. The ability to rapidly identify tumor mutations by next-generation sequencing and to predict with a high level of accuracy which mutations can induce cell-mediated immunity, has prompted the development of personalized cancer vaccines constituted by neo-epitopes administered either as synthetic peptides or RNAs [11]. It has recently been shown that BALB/c and C57BL/6 mice challenged with CT26 and B16F10 tumor cell lines, respectively, and subsequently immunized with RNAs encoding tumor-specific neo-epitopes did not develop tumors [11,12]. Moreover, melanoma and glioblastoma patients treated with neo-epitope-based personalized vaccines showed objective clinical responses, which also appeared to synergize with checkpoint inhibitor therapy [13,14,15].

Considering its ability to enhance the antitumor T cell population [5], the gut microbiome could also influence the efficacy of neo-epitope-based cancer vaccines by either enhancing the frequency of vaccine-induced T cells or by complementing their activity with the elicitation of additional T cells recognizing other cancer epitopes. However, no data on the role of gut microbiome in cancer vaccines have been reported so far.

In this work, we have investigated whether and to what extent the gut microbiome can influence the antitumor activity of neo-epitope-based cancer vaccines. To address this question we have used the BALB/c-CT26 murine tumor model and we have tested whether the efficacy of a vaccine constituted by OMVs decorated with five CT26-specific neo-epitopes [12,16] could be potentiated by *Bifidobacterium* administration during vaccination. Here, we show that *Bifidobacterium* administration per se has a beneficial effect on tumor growth inhibition. Furthermore, the combination of *Bifidobacterium* administration and vaccination results in a protection which is statistically superior to vaccination alone and to *Bifidobacterium* administration alone. The beneficial effect of Bifidobacterium on vaccine correlates with an increase in the frequency of vaccine-specific T cells. Our gut microbiome analysis by 16S rRNA gene sequencing and shotgun metagenomics shows that *Bifidobacterium* does not efficiently colonize murine intestine but influences the way tumor growth alters the gut microbiome composition, leading to the identification of *Muribaculaceae* and *Lachnospiraceae* as possible antitumorigenic and protumorigenic species, respectively.

Altogether, our data strengthen the importance of the microbiome in cancer immunotherapy, and suggest that for its optimal efficacy, cancer vaccination should be combined with microbiome-based antitumor strategies.

## 2. Materials and Methods

### 2.1. Bacterial Strains, Cell Lines and Animals

Plasmid assembly was previously described by Grandi et al. [16] and the polymerase incomplete primer extension (PIPE) method [17] was performed in *E. coli* HK-100 strain [16]. OMVs were purified from *E. coli* BL21(DE3)Δ*ompA* strain as previously described [16,18,19]. *E. coli* was grown in lysogeny broth (LB broth medium Merck, Darmstadt, Germany) at 30 °C and/or 37 °C and 180 rpm. When required, Ampicillin (Amp, Carl Roth, Karlsruhe, Germany) was added to a final concentration of 100 μg/mL. The CT26 cell line was obtained from ATCC and cultured in complete RPMI (RPMI 1640 (Gibco, Thermo Fisher Scientific, Waltham, MA, USA) supplemented with 10% Fetal Bovine Serum (FBS, Sigma-Aldrich), with 100 units/mL of penicillin and 100 µg/mL of streptomycin (Gibco, Thermo Fisher Scientific, Waltham, MA, USA) and 2 mM of L-glutamine (Gibco, Thermo Fisher Scientific, Waltham, MA, USA), and grown at 37 °C in 5% CO_2_. Mice were challenged with mycoplasma-negative cells.

4–8-week-old female BALB/c mice were obtained from Charles River Laboratories. Mice experiments were performed in compliance with experimental protocols approved by the Animal Ethical Committees of University of Trento (Trento, Italy), Toscana Life Sciences (Siena, Italy) and by the Italian Ministry of Health. Mice were caged in groups of 5 in ventilated cages. Treatments were randomly assigned to mice, and mice within the same cage received the same therapy.

### 2.2. Synthetic Peptides

The synthetic peptides were obtained from GenScript in lyophilic form and dissolved in sterile deionized water at final concentration of 5 mg/mL. Peptide names and corresponding amino acid sequences are listed in Table 1:

### 2.3. OMV Engineering with CT26-Derived Neo-Epitopes

Selected CT26 neo-epitopes were expressed in BL21(DE3)Δ*ompA* strain as C-terminal fusions as previously described [16]. Decorated OMVs were purified from recombinant strains expressing fusion proteins that were grown at 30 °C and 180 rpm in LB medium (starting OD_600_ = 0.1). At an OD_600_ = 0.5, protein expression was induced by the addition of 0.1 mM Isopropyl β-d-1-thiogalactopyranoside (IPTG, Merck, Darmstadt, Germany). After 4 h, culture supernatants were separated from living bacterial cells by a centrifugation step at 6000× *g* for 15 min followed by a filtration through a 0.22 μm-pore-size filter (Merck, Darmstadt, Germany). Supernatants were then subjected to high-speed centrifugation (200,000× *g* for 2 h) and pellets containing the OMVs were finally resuspended in sterile PBS. OMV amounts were estimated by measuring protein concentration using DC protein assay (Bio-Rad Laboratories, Hercules, CA, USA). Then, 10 or 20 µg (protein content) were resuspended in sodium dodecyl sulfate-polyacrylamide gel electrophoresis (SDS-PAGE) Laemmli buffer and heated at 100 °C for 10 min. Any kD™ Criterion™ TGX Stain-Free™ Protein Gels (Bio-Rad Laboratories, Hercules, CA, USA) were used in TrisGlicyne buffer (Bio-Rad Laboratories, Hercules, CA, USA) to separate proteins, which were finally revealed by Coomassie Blue staining (ProBlue Safe Stain, Giotto Biotech, Sesto Fiorentino, Italy).

### 2.4. Animal Studies

Animal experiments were carried out in accordance with experimental protocols reviewed and approved (1060/2016-PR and 1153/2020-PR) by the Animal Ethical Committees of University of Trento (Trento, Italy) and Toscana Life Sciences (Siena, Italy) and by the Italian Ministry of Health.

Mice were examined two times a day to check for signs of pain and distress according to humane endpoints. Animals showing abnormal respiration rate and posture, and loss of weight (more than 20%) were anesthetized and subsequently sacrificed with CO_2_ followed by cervical dislocation, as specified in the approved experimental protocols.

Tumor challenge—For cancer immunotherapy studies, two and four independent experiments with 5 mice/group were performed for each vaccination treatment and controls, respectively (control groups *n* = 20, vaccinated groups *n* = 10). First, 1.5 × 10^5^ CT26 cells were subcutaneously injected into BALB/c mice on day 0. Mice were then immunized intraperitoneally on day 1, 4, 8, 11, 15, (18) and 22 with either 20 µg of ads-pentatope OMVs (20 µg of empty OMVs adsorbed to 20 µg of each one of the M03, M20, M26, M27 and M68 peptides), 20 µg of pentatope-engineered OMVs (4 µg of each one of FhuD2-M03, FhuD2-M20, FhuD2-M26, FhuD2-M27 and FhuD2-M68 OMVs) or 20 µg of Empty OMVs (negative control). *Bifidobacterium* cocktail (*B. bifidum*, *B. longum, B. lactis* and *B. breve*) was purchased from Seeking Health, LLC (Bellingham, WA, USA) and was resuspended in sterile PBS at 5 × 10^9^ CFU/mL. On day 7 and 14 post CT26 cell injection, mice were given either 1 × 10^9^ CFUs of the *Bifidobacterium* cocktail or PBS (negative control), by a 200 µL gavage, as previously described [5]. Tumor growth was measured every 2–4 days from day 11. At the end of the challenge experiment, spleens were collected from euthanized mice and analyzed as described below. No animal was excluded during or at the end of the experiment. 

Fecal samples were collected 7 and 3 days before tumor cell inoculation to determine mice baseline microbiota. Feces were also collected on day 1 post tumor inoculation and before the first immunization with OMVs to test the effect of tumor cell injection on the microbiota. Further fecal samples were harvested on day 7 before the first gavage, on day 10, on day 18 post tumor inoculation and at the end of the challenge experiment.

Tumor volume was calculated with the formula (A × B^2^)/2, where A was the longest and B the shortest diameter of the tumor, respectively. Measures were taken with a caliper by an unblinded operator. Statistical analysis (ANOVA and unpaired, one-tailed Student’s *t*-test) and graphs were processed using GraphPad Prism 5.03 software.

*T cell analysis*—T cell analysis was performed with minor modifications as previously described [16,19]. At the end of the tumor challenge, spleens were homogenized and splenocytes filtered through a 70 μm cell Strainer (Becton Dickinson, BD Franklin Lakes, NJ, USA). After centrifugation at 400× *g* for 7 min, splenocytes were resuspended in complete RPMI (Gibco, Thermo Fisher Scientific, Waltham, MA, USA) and aliquoted in a 96-well plate (Corning Incorporated, Corning, NY, USA) at a concentration of 1 × 10^6^ cells per well. Cells were stimulated with 10 μg/mL of each one of the 5 pentatope peptides (M03, M20, M26, M27 and M68). As positive and negative controls, splenocytes were stimulated with phorbol 12-myristate 13-acetate (PMA, 0.5 μg/mL, Merck, Darmstadt, Germany) and ionomycin (1 μg/mL, Merck, Darmstadt, Germany) or with 10 μg/mL of an unrelated peptide, respectively. After 2 h of stimulation at 37 °C, Golgi Stop solution (Becton Dickinson, BD Franklin Lakes, NJ, USA) was added to each well for 4 h at 37 °C. After two washes with PBS, LIVE/DEAD™ Fixable Near-IR Dead Cell Stain Kit (Thermo Fisher Scientific, Waltham, MA, USA) was incubated with the splenocytes for 20 min at room temperature in the dark. After two washes with PBS and permeabilization and fixing with Cytofix/Cytoperm (Becton Dickinson, BD Franklin Lakes, NJ, USA) following the manufacturer’s protocol, Fc receptors were blocked with α-CD16/CD32 for 15 min at room temperature. Splenocytes were stained with the following fluorescent-labeled antibodies: α-CD3-APC (BioLegend, San Diego, CA, USA), α-CD4-BV510 (BioLegend, San Diego, CA, USA), α-CD8-PECF594 (Becton Dickinson, BD Franklin Lakes, NJ, USA), and α-IFN-γ-BV785 (BioLegend, San Diego, CA, USA). Samples were acquired on a BD LSRII flow cytometer. Briefly, after gating live lymphocytes from all events, single cells were selected on SSC-A and SSC-W, excluding both duplets and aggregates. T lymphocytes were then selected for CD3, CD4 and CD8 expression. IFN-γ-positive T cells were finally visualized and calculated as percentage of IFN-γ/CD4 and IFN-γ/CD8 double-positive cells out of the total CD4 or CD8 cells, respectively. Graphs and statistical analyses (unpaired, one-tailed Student’s *t*-test) were performed with GraphPad Prism 5.03.

### 2.5. Fecal DNA Extraction and 16S Metagenomic Sequencing Libraries Preparation

Microbiota DNA was extracted from feces (fecal pellets were collected from each mouse) using the DNeasy PowerSoil Kit (Qiagen, Hilden, Germany) following manufacturer’s instructions and quantified using the Qubit 4 fluorometer (Thermo Fisher Scientific). Next, 16S rRNA gene sequencing libraries were generated following Illumina’s “16S Metagenomic Sequencing Library Preparation guide” (Illumina, San Diego, CA, USA). Amplicons of approximately 460 nucleotides were generated by amplifying the DNA extracted from the mice feces with primers 16S Amplicon F 5′- TCGTCGGCAGCGTCAGATGTGTATAAGAGACAGCCTACGGGNGGCWGCAG-3′ and 16S Amplicon R 5′-GTCTCGTGGGCTCGGAGATGTGTATAAGAGACAGGACTACGVGGGTATCTAA TCC-3′, targeting the V3 and V4 variable regions of the 16S rRNA gene. Primers contained 5′ overhang adapters needed for the following library preparation. Free primers and primer dimers were removed using AMPure XP beads as recommended in the guide. Next, dual indices and Illumina sequencing adapters were attached to the PCR amplicons using the Nextera XT Index Kit (Illumina, San Diego, CA, USA) following manufacturer’s instructions, and a clean-up of the libraries was performed using the AMPure XP beads. Library quality was assessed using the Caliper LabChip GX (High-Throughput Bioanalyzer, PerkinElmer, Waltham, MA, USA) according to the manufacturer’s instructions. Normalizations were then performed as described in the Nextera XT “DNA Library Prep Kit” to ensure an equal representation of each one in the final pool, obtained by mixing equal volumes of normalized libraries in a single tube. The sequencing was performed on the MiSeq platform (Illumina, San Diego, CA, USA) using the paired-end 600-cycle flow cell to allow the longest read lengths.

### 2.6. 16S rRNA Sequences Processing and Analysis

Paired-end reads were joined together and processed for quality filtering using pandaseq [20], with default parameters. Open reference Operational Taxonomic Unit (OTU)-picking was performed in QIIME v1.9 [21] by clustering with uclust [22] at 97% sequence similarity threshold. OTUs were taxonomically annotated with RDP [23] using SILVA 132 [24] as reference. A total of 37,415 OTUs with more than 10 sequences were retained and used for further analyses.

Alpha diversity was estimated with Chao1, Simpson, Shannon and observed number of OTUs and calculated through the “alpha_diversity” function, as implemented in R package Phyloseq [25]. Difference in alpha diversity was tested with a two-tailed *t*-test. Beta diversity was explored by using multidimensional scaling (MDS) on a distance matrix calculated with the Weighted UniFrac measure [26]. Permutational analysis of variance (PERMANOVA) [27] as implemented in vegan “adonis” function was used to test differences in microbiome composition between groups and performed with 999 permutations. Differentially abundant species were discovered using a Wilcoxon test between T3 and T7 and selected based on a false discovery rate (FDR) less than 0.05 [28].

Spearman’s correlation analysis was performed to correlate *Muribaculaceae* relative abundance between T3 and T7 and the extent of the tumor mass. 

Where alpha and beta diversity and correlation analysis were concerned, the significance threshold was set at *p* < 0.05. For the differential abundance analysis, False Discovery Rate (FDR, *q*-value 39) less than 0.05 was considered statistically significant [28].

### 2.7. Shotgun Metagenomic Analysis

Sequencing libraries were prepared using the Illumina^®^ DNA Prep, (M) Tagmentation kit (Illumina, San Diego, CA, USA), following the manufacturer’s guidelines. A cleaning step on the pool with 0.6x Agencourt AMPure XP beads was implemented. Sequencing was performed on a Novaseq600 S4 flow cell (Illumina) at the internal sequencing facility at University of Trento, Trento, Italy. Metagenomic shotgun sequences were quality-filtered using trim galore discarding all reads of quality <20 and shorter than 75 nucleotides. Filtered reads were then aligned to the human genome (hg19) and the PhiX genome for human and contaminant DNA removal using Bowtie 2 [29], yielding an average of 40 million bases in high-quality reads in each sample. Species-level microbial abundances were obtained through the bioBakery suite of tools using MetaPhlAn v.4.0 with default settings (database January 2021) [30]. Relative abundances at species level were analyzed in R. Each metagenomic sample was assembled using MEGAHIT v1.1.1 [31]. Only the assembled contigs ≥1.5 kilobases (kb) have been selected for the binning step with MetaBAT2 v.2.12.1 [32] using the parameter m 1500. Quality control of the assemblies was performed using CheckM v.1.1.3 [33] using default parameters.

Beta diversity was explored using multidimensional scaling (MDS) on a distance matrix calculated with the Bray–Curtis distance. Differentially abundant species were discovered using a Wilcoxon test between T3 and T7 and selected based on a false discovery rate (FDR) less than 0.05. Spearman’s correlation analysis was performed between differences in relative abundances at T7 and T3 and the extent of the tumor volume.

### 2.8. Analysis of CT26 Epitopes in Microbiomes

Analysis of CT26 epitopes within microbiomes was performed by selecting the four CT26 CD8 epitopes described by Kreiter et al. [12] and mapping these epitopes against metagenomic reads using DIAMOND blastx (v0.9.30.131) [34]. All resulting MMEs carrying the CT26 tumor mutation were filtered and used as input for the prediction of peptide/MHCI binding affinity. The resulting MMEs of lengths 8,9 and 10 were prioritized according to their peptide/MHCI binding affinity (H-2-Dd, H-2-Kd and H-2-Ld) predicted using NetMHC, NetMHCpan, PickPocket, SMM and SMMPMBEC integrated into pVACbind [35]. Only mutated MMEs with a percentile rank less than 0.5, lower than the respective wild-type epitope, with an identity higher than 60% with the corresponding CD8 CT26 neo-epitope and present in at least five mice were selected. To understand in which species the resulting MMEs were present, each MME was mapped against all metagenomes assembled using DIAMOND blastx (v0.9.30.131) [34], and only species in which at least one MME was found with a percentage identity equal to 100% were considered.

## 3. Results

### 3.1. Cooperative Antitumor Effect of Cancer Neo-Epitope Vaccination and Bifidobacterium Administration

**Figure 1:**
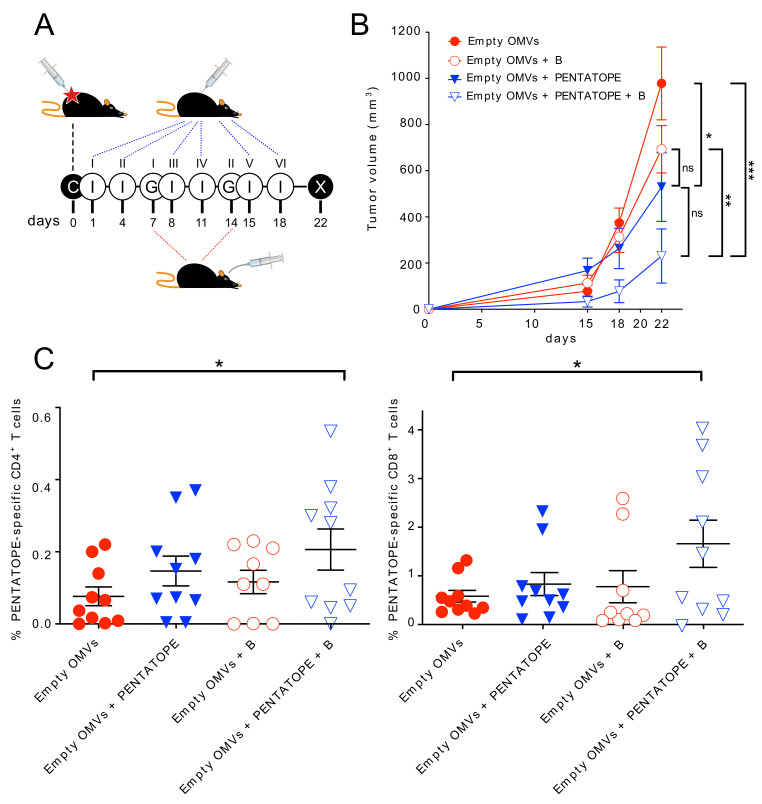
Effect of peptide-adsorbed OMV vaccination and Bifidobacterium administration on tumor growth inhibition. (**A**) Schematic representation of immunization schedule. Four groups of BALB/c mice (5 mice/group, two independent experiments) were injected with 1.5 × 10^5^ CT26 cells (“C”) into the right flank. The day after challenge, two groups were vaccinated (“I”) every three days with 20 μg OMVs while the other two groups were injected with OMVs + pentatope (20 μg OMVs + M03, M20, M27, M68, M26 peptides, 20 μg each). At day 7 and 14 from tumor challenge, mice from one group immunized with OMVs and one group immunized with OMVs + Pentatope were given two oral gavages (“G”) of Bifidobacterium cocktail. X, end of the experiment. (**B**) Analysis of tumor development. The tumor growth in mice treated as described in (**A**) was followed by measuring the tumor volume (mean ± s.e.m.) over a period of 22 days from challenge. (**C**) Frequency of pentatope-specific CD4^+^ and CD8^+^ T cells. At the end of the experiment, spleens were collected from each mouse and the frequency of IFN-γ-producing, pentatope-specific CD4^+^ and CD8^+^ T cells was determined by flow cytometry after splenocyte stimulation with pentatope peptides (see Materials and Methods for details). Statistical analysis was performed using ANOVA and unpaired, one-tailed Student’s *t*-test. * *p* < 0.05; ** *p* < 0.01; *** *p* < 0.001. ns, not significant.

Sivan and coworkers showed that C57BL/6 mice from the Taconic Farms (TAC)TAC mice were partially protected from melanoma B16 cells when animals were given two oral gavages of a cocktail of *Bifidobacterium* species (*B. bifidum*, *B. longum*, *B. lactis* and *B. breve*) after tumor challenge. Since BALB/c mice developed large tumors when CT26 cells are injected subcutaneously (s.c.) [16], we first asked the question as to whether the same Bifidobacterium cocktail used by Sivan and coworkers could have an effect on CT26-induced tumor growth in BALB/c mice. To this aim, mice were given 1.5 × 10^5^ CT26 cells s.c., and after seven days the animals received 1 × 10^9^ CFUs of *Bifidobacterium*. The oral gavage was repeated after one week and tumor growth was monitored for a period of 22 days from challenge (Figure 1A). As shown in Figure 1B, *Bifidobacterium* administration partially delayed tumor growth.

Next, we investigated whether the administration of the *Bifidobacterium* cocktail could synergize with neo-epitope-based vaccination. To this aim, we took advantage of our previous work in which we showed that the immunization of BALB/c mice with bacterial Outer Membrane Vesicles (OMVs) decorated with five CT26 immunogenic epitopes described by Kreiter and coworkers [12] (four CD4+ T cell epitopes (M03, M20, M27 and M68) and one CD8+ T cell epitope (M26)) partially protected mice from tumor growth [16]. Synthetic peptides, 25 amino acids long, encompassing the five CT26-specific neo-epitopes were mixed (20 μg each) with 20 μg of OMVs from *E. coli* BL21(DE3)*ΔompA* and after tumor challenge the peptide-adsorbed OMVs were used to immunize mice in the presence or absence of *Bifidobacterium* administration (Figure 1A). Vaccination partially inhibited tumor growth (*p*-value 0.0265), and the coadministration of pentatope-adsorbed OMVs and *Bifidobacterium* further reduced tumor development (*p*-value 0.0007). Importantly, the difference in tumor inhibition was also statistically significant when the pentatope-adsorbed OMVs + *Bifidobacterium* treatment is compared with empty OMVs + *Bifidobacterium* treatment (*p*-value 0.0041). The statistical difference among the four groups was also confirmed by applying the ANOVA test (*p*-value 0.0033) (Figure 1B). 

Since the administration of *Bifidobacterium* to C57BL/6 mice challenged with B16F10 cells promoted the elicitation of cancer-specific effector T cells [5], we also investigated whether a similar effect occurred in the BALB/c-CT26 model, focusing our attention on T cells specifically recognizing the M03, M20, M26, M27 and M68 neo-epitopes. At the end of the experiment reported in Figure 1A,B, spleens were collected, splenocytes stimulated with the mixture of the five synthetic peptides corresponding to M03, M20, M26, M27 and M68 neo-epitopes, and IFN-γ-producing CD4^+^ and CD8^+^ T cells analyzed by flow cytometry. As far as vaccine-specific CD4^+^ T cell epitopes are concerned, they were induced in all groups, even though to a relatively low frequency, including OMVs alone. This is consistent with the fact that OMVs have built-in adjuvanticity and that neo-epitopes are expected to be expressed during tumor progression. The frequency of such T cell populations were increased when synthetic peptides carrying the neo-epitopes were “adsorbed” to the OMVs. *Bifidobacterium* alone was also capable of stimulating some epitope-specific CD4^+^ T cells, whose frequency was increased when *Bifidobacterium* administration and vaccination were combined (Figure 1C). With respect to CD8^+^ T cells, vaccination alone and *Bifidobacterium* administration alone induced epitope-specific CD8^+^ T cells, though their frequency was not statistically different from the control group. By contrast, the frequency increased with the combination of vaccination and *Bifidobacterium*. Since the neo-epitope vaccine included only one CD8^+^ T cell epitope (M26) [12], these data indicate that *Bifidobacterium* administration was capable of specifically amplifying the M26 CD8^+^ T cell population.

**Figure 2:**
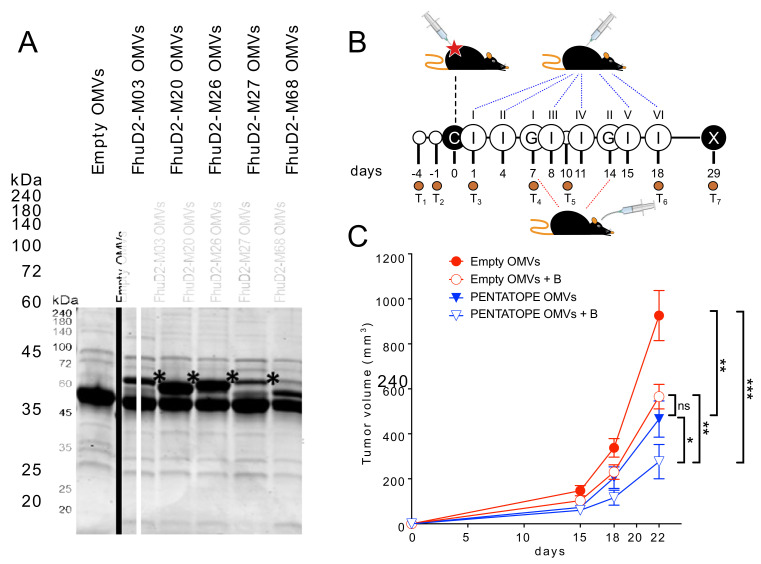
Effect of engineered OMV vaccination and Bifidobacterium administration on tumor growth inhibition. (**A**) SDS-PAGE analysis of engineered OMVs. M03, M20, M27, M68, M26 peptides were fused to the C-terminus of FhuD2 and the plasmids expressing the fusion proteins were used to transform *E. coli* BL21(DE3)ΔompA strain. OMVs were purified from the supernatant of the recombinant clones and 20 μg of each OMV preparation were analyzed by SDS-PAGE. Stars indicate the bands corresponding to each fusion protein. (**B**) Schematic representation of animal treatment. Four groups of BALB/c mice (5 mice/group, two independent experiments) were injected with 1.5 × 10^5^ CT26 cells (“C”) into the right flank. The day after challenge, two groups were vaccinated (“I”) every three days with 20 μg of OMVs while the other two groups were injected with a mixture (4 μg each) of the five engineered OMVs (20 μg in total). At day 7 and 14 from tumor challenge, mice from one group immunized (“I”) with OMVs and one group immunized with engineered OMVs were given two oral gavages of Bifidobacterium cocktail. T1-7 correspond to the days when fecal samples were collected for microbiome analysis (see text and Figure 3). X, end of the experiment. (**C**) Analysis of tumor development. The tumor growth in mice treated as described in (**A**) was followed by measuring the tumor volume (mean ± s.e.m.) over a period of 22 days from challenge. Statistical analysis was performed using ANOVA and unpaired, one-tailed Student’s *t*-test. * *p* < 0.05; ** *p* < 0.01; *** *p* < 0.001. ns, not significant.

To confirm the cooperative protective activity of the neo-epitope-based vaccine and *Bifidobacterium,* a second experiment was carried out in which the peptide-adsorbed vaccine was replaced with a vaccine formulation comprising a combination of engineered OMVs, where each was decorated with one of the five selected T cell epitopes. OMV engineering was accomplished by fusing the epitopes to the C-terminus of lipidated FhuD2, which ist a *Staphylococcus aureus* antigen we previously showed to compartmentalize in OMVs and to be exposed on the membrane surface [16]. All five neo-epitopes were successfully incorporated into the OMVs and constituted a significant fraction of their total protein content (Figure 2A). First, we confirmed that engineered OMVs elicit pentatope-specific CD4^+^ and CD8^+^ T cells (Figure S1). 

Then, we challenged mice with CT26 cells, and we combined the immunization with engineered OMVs with *Bifidobacterium* administration (Figure 2B). In line with what obtained with the first experiment, the average tumor volumes were significantly different among the four groups (ANOVA *p*-value < 0.0001). Moreover, vaccination and *Bifidobacterium* administration reduced tumor growth to a level significantly higher than vaccination alone and *Bifidobacterium* alone (empty OMVs vs. pentatope OMVs + *Bifidobacterium*: *p*-value 0.0002; pentatope OMVs vs. pentatope OMVs + *Bifidobacterium*: *p*-value 0.0495) (Figure 2C).

### 3.2. The Gut Microbiome Is Reshaped throughout the Animal Treatment

In addition to following the effect of vaccination and *Bifidobacterium* administration on tumor growth, we also wanted to investigate whether and to what extent the composition of the gut microbiome could have been modified by tumor challenge, vaccination and *Bifidobacterium* administration. To this aim, fecal samples were collected before and after tumor challenge and at different time points throughout the vaccination schedule and *Bifidobacterium* administration (Figure 2B) for subsequent metagenome analysis. Fecal samples were collected twice before tumor challenge (T1 and T2), one day after tumor challenge (T3), and four times during immunizations and oral gavages (T4–T7) (Figure 2B). Total DNA was extracted from the fecal samples collected from each individual mouse and the gut microbiome was characterized via 16S rRNA gene sequencing (see Materials and Methods).

**Figure 3:**
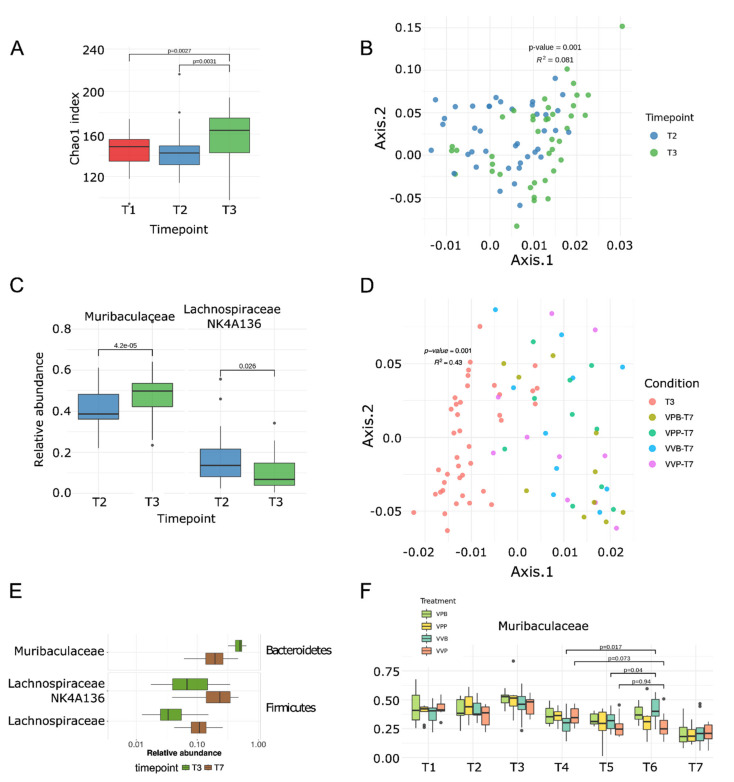
Microbiome variations identified by 16s rRNA sequencing. (**A**–**C**) Alteration of microbiome composition at tumor injection (T1/T2 vs. T3) as measured by alpha diversity index Chao1 (**A**), multidimensional scaling (MDS) on samples using the Weighted UniFrac measure (*p* = 0.001, PERMANOVA with 999 permutations) (**B**) and relative abundance distribution of key bacteria (**C**). This later analysis shows an increase in relative abundance of bacteria belonging to the *Muribaculaceae* group (*q*-value = 4.2 × 10^−5^ Wald test) and a decrease in the genus *Lachnospiraceae NK4A136* (*q*-value = 0.026 Wald test). (**D**–**E**) Comparison of gut microbiome in mice at T3 and T7. Multidimensional scaling (MDS) on samples using the Weighted UniFrac measure showed a statistically significant difference (*p* = 0.001, PERMANOVA with 999 permutations). (**D**) Differentially abundant species between T3 and T7 (Wilcoxon test). Only species found to be significantly differentially abundant after false discovery-rate error correction (*p_adj_* < 0.05) and with a median relative abundance >0.01 are reported. (**E**,**F**) Boxplot of relative abundances of *Muribaculaceae* across timepoints and separated by treatment. *Muribaculaceae* was altered after administrating the *Bifidobacterium* in mice vaccinated with nondecorated vesicles. VPB: Pentatope OMVs + *Bifidobacterium* gavage; VPP: pentatope OMVs; VVB: empty OMVs + *Bifidobacterium* gavage; VVP: empty OMVs.

We first found that the gut bacterial population was substantially modified within a day of tumor challenge. Alpha diversity Chao1 index increased soon after the tumor challenge compared to the baseline samples (T3 vs. T1, *p* = 0.0027 and T3 vs. T2, *p* = 0.0031; Figure 3A). The overall composition of the microbiome, calculated through the Weighted Unifrac as a beta-diversity measure, was also profoundly modified between T2 and T3 time points (i.e., pre and post tumor challenge; PERMANOVA *p* = 0.001; Figure 3B). The two species that were most responsible for this shift were *Muribaculaceae* (previously called *Bacteroidales S24-7*, the most abundant bacterial family in these mice) and *Lachnospiraceae NK4A136*. While the relative abundance of *Muribaculaceae* increased after tumor challenge (FDR-corrected *q*-value = 4.2 × 10^−5^, from average 40% s.d. 9.8% at T2 to average 48% s.d. 10.6% at T3, Figure 3C), *Lachnospiraceae NK4A136* markedly decreased from T2 to T3 (*q*-value = 0.026, from average 16.2% s.d. 11.6% at T2 to average 9.7% s.d. 7.9% at T3, Figure 3C).

The microbiome further changed dramatically with the progression of the experiment (Figure S2). The same bacteria that were already altered after tumor challenge were the ones most affected with an inverse trend throughout the experiment as the tumor volume increased. This was particularly evident from the separation of the T3 and T7 time points based on their between-samples distances (Weighted Unifrac) (Figure 3D). Taking all groups into account, a total of 89 species showed a different abundance between T3 and T7 (Figure S3). In particular, the relative abundances of *Muribaculaceae* were drastically reduced at the end of the experiment (T7) compared to those after the tumor challenge (T3, *q*-value = 2.2 × 10^−5^, from average 48% s.d. 10.6% at T3 to average 21.1% s.d. 9.97% at T7, Figure 3E), reaching levels substantially lower than the baseline at T1 and T2 (Figure 3F). On the other hand, *Lachnospiraceae NK4A136* (*q*-value = 6.3 × 10^−9^) and *Lachnospiraceae* (*q*-value = 3.5 × 10^−12^) relative abundance was higher at T7 than at T3, and such changes appeared to parallel to the tumor growth (Figure 3E). 

As previously observed, mice that received both pentatope OMVs and *Bifidobacterium* administration showed a significantly reduced tumor development (Figure 2C). Interestingly, although *Bifidobacterium* spp. were not detected in our analysis, at least not at a level higher than the detection limits of our experimental conditions (>0.002% relative abundance, see Material and methods), the *Bifidobacterium* administration specifically favored the expansion of *Muribaculaceae*. Moreover, the administration of *Bifidobacterium* was the only treatment able to invert the trend of *Muribaculaceae* decrease. *Muribaculaceae* relative abundances at T6 (i.e., after two *Bifidobacterium* gavages) were significantly higher than those at T4 in both groups receiving the *Bifidobacterium* administration, and significantly lower in both groups without such treatment (Figure 3F).

**Figure 4:**
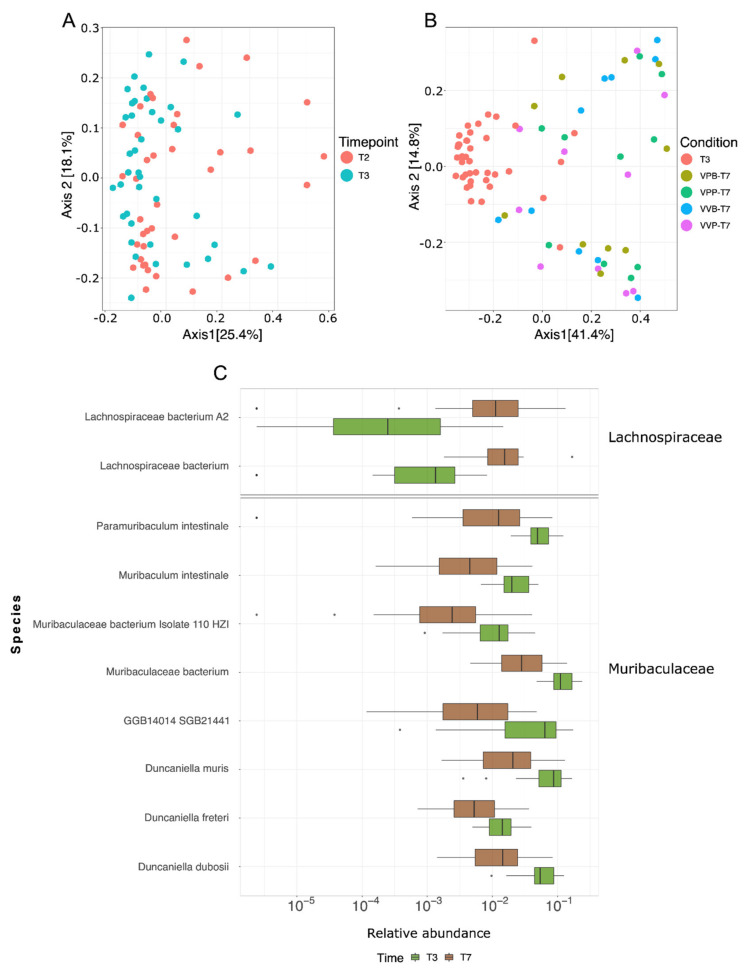
Microbiome variations identified by shotgun metagenomic sequencing. (**A**) Alteration of microbiome composition at tumor injection (T1/T2 vs. T3) as measured by multidimensional scaling (MDS) on samples using Bray–Curtis distance. (**B**) Comparison of gut microbiome in mice at T3 and T7. Multidimensional scaling (MDS) on samples using Bray–Curtis distance. (**C**) Differentially abundant species between T3 and T7 (Wilcoxon test). Only species found to be significantly differentially abundant after false discovery rate error correction (*p_adj_* < 0.05) and with a median relative abundance > 0.01 are reported. VPB: Pentatope OMVs + *Bifidobacterium* gavage; VPP: pentatope OMVs; VVB: empty OMVs + *Bifidobacterium* gavage; VVP: empty OMVs.

Having demonstrated by 16S rRNA sequencing that *Bifidobacterium* administration altered gut microbiome composition, we decided to improve the granularity of our analysis by performing shotgun metagenomic sequencing of the same fecal DNAs (see Materials and Methods for details). The sequence analysis confirmed that the tumor challenge altered the composition of the gut microbiome (i.e., pre and post tumor challenge; PERMANOVA *p* = 0.024, Figure 4A) and that the gut microbiome changed substantially over time (PERMANOVA *p* = 0.001, T7 vs. T3, Figure 4B) as demonstrated by their between-samples distances measured as beta diversity (Bray–Curtis distance). Thanks to the higher resolution of the shotgun sequencing with respect to 16S rRNA gene sequencing, 45 bacterial species within *Muribaculaceae* and *Lachnospiraceae* families were found to be significantly modified from T3 to T7 (Figure S4) (Wilcoxon test, *p_adj_* < 0.05), and the most affected species are reported in Figure 4C. Interestingly, the relative abundance of a few species appeared to parallel the kinetics of tumor. These data indicate the existence of species which vary as a result of the different treatments and have an opposite effect on tumor growth.

## 4. Discussion

The role of the gut microbiome in cancer immunity is now supported by a large body of evidence both in mice and humans. Tumor growth in the same inbred mice challenged with syngeneic cancer cell lines is profoundly affected by their gut microbiome profiles, which can differ due to housing conditions, antibiotic treatment or oral administration of microbial cocktails [3,5]. Importantly, a large epidemiological study in humans involving 125,441 cases and 590,510 matched controls has suggested that antibiotic exposure may affect the frequency of lung, prostate and bladder cancer [36].

It is also becoming evident that the gut microbiome can substantially influence the effectiveness of cancer immunotherapies. Such influence has been so far demonstrated for two immunotherapeutic strategies, namely, administration of checkpoint inhibitors and adoptive T cell therapy (ACT). As far as checkpoint inhibitors are concerned, it has been shown in human patients that responders and nonresponders to the therapy can be stratified on the basis of their microbiome, with some species positively and some species negatively correlating with the therapeutic effects [37,38]. Similar results have been observed in cancer mouse models. Gut microbiome dysbiosis abolishes the anticancer activity of anti-CTLA4 and anti-PD-1/PD-L1 antibodies, and fecal transplantation from responding animals and even from responding patients re-establish the beneficial effects of checkpoint inhibitors [39].

More recently, using a mouse model in which animals were challenged with TC1 cell line (cervix and lung tissue culture number 1) expressing HPV E6 and E7 viral oncogenes, it was demonstrated that the gut microbiome also affects the efficacy of ACT. Adoptive transfer of T cells specific for HPV E6 and E7 antigens substantially protected mice from TC1 challenge. However, such protection was markedly reduced if mice were previously treated with vancomycin, an antibiotic against Gram-positive bacteria that is not absorbed and therefore is mostly active on gut microbiota [40].

With this work we have further expanded the evidence of the role of the gut microbiome in cancer immunotherapy by demonstrating in a mouse model that the effectiveness of a neo-epitope-based cancer vaccine could be potentiated by altering the gut microbiome with oral gavages of *Bifidobacterium*. We showed that the administration of the same *Bifidobacterium* cocktail previously used by Sivan et al. to potentiate PD-L1 therapy [5] was sufficient to enhance the antitumor activity of a cancer vaccine comprising OMVs formulated with five mutation-derived neo-epitopes. In particular, we showed that BALB/c mice challenged with CT26 cells were partially protected by repeated immunizations with the neo-epitope-OMV vaccine, and that such protection was enhanced by two oral administrations of *Bifidobacterium*. *Bifidobacterium* did not colonize the intestine as revealed by 16S RNA gene sequence analysis and shotgun metagenomic sequencing of total DNA extracted from fecal samples. However, *Bifidobacterium* administration did alter the composition of the gut microbiome. In particular, *Bifidobacterium* favored the increase in *Muribaculaceae*. Interestingly, since our data indicate that tumor growth directly correlated with a decrease in *Muribaculaceae*, as well as other bacterial species (Figure 3E–F, Figure 4C and Figure S4), it appears that *Bifidobacterium* exerted at least part of its antitumor effect by counterbalancing the perturbation of the gut microbiome caused by tumor growth. In terms of immune responses, we focused our attention on analyzing how the frequency of the vaccine-specific T cells could have been affected by the *Bifidobacterium* administration. Interestingly, we found an increase in both CD4^+^ and CD8^+^ T cells, but such an increase was particularly pronounced and statistically significant in the case of CD8^+^ T cells. Since our neo-epitope vaccine included only one CD8^+^ T cell epitope (M26), these results suggest that M26 is specifically involved in tumor cytotoxicity.

We acknowledge that while our data demonstrate that the efficacy of neo-epitope vaccines is influenced by the gut microbiome, and highlight the importance of combining vaccination with the administration of a proper cocktail of commensal bacteria, a multitude of questions remain to be addressed. 

First, our *Bifidobacterium* administration regime was based on two administrations given one week apart, the first starting seven days from tumor challenge. This was the same schedule used by Sivan et al. to potentiate PD-L1 antitumor effect in a C57BL/6 model [5]. It would be interesting to test whether more-frequent administrations would promote *Bifidobacterium* colonization and/or would maintain the levels of “beneficial species” sufficiently highly throughout the experiment, to further inhibit tumor progression. For instance, in a recent publication, Lee and coworkers demonstrated the potentiation of PD-1 therapy when mice were repeatedly given oral administration of *Bifidobacterium bifidum* strain, starting two weeks before tumor challenge [6].

Second, we have so far tested the effect of *Bifidobacterium* administration and vaccination using only the BALB/c-CT26 tumor model. As already pointed out, the first evidence that *Bifidobacterium* positively influenced immunotherapy was reported by Sivan et al. in the C57BL/6-B16.SIY model. In this model, the authors showed that *Bifidobacterium* did colonize the intestine, and the level of colonization nicely correlated with the frequency of tumor-specific T cell infiltration. Interestingly, the authors also showed that the level of *Muribaculaceae* had a tendency to increase upon oral administration of *Bifidobacterium*, although not in a statistically significant manner. Therefore, it would be relevant to see whether the synergistic activity of *Bifidobacterium* and neo-epitope vaccination would also be observed in this model. A few B16.SIY-specific immunogenic neo-epitopes to be used in vaccine formulations have been described and therefore these experiments would be technically feasible.

A third important question, is whether and to what extent the synergistic activity of gut microbiome and vaccination is influenced by the selection of epitopes included in the vaccine formulation. In this work, we tested five out of several CT26-specific CD4^+^ and CD8^+^ T cell epitopes [12]. In particular, our neo-epitope cocktail includes only one CD8^+^ T cell epitope, and based on our data as well as already-published data [5,15,39], CD8^+^ T cells seem to be particularly influenced by variations in the gut microbiome. Therefore, it would be interesting to repeat the experiments here described using vaccines formulated with different neo-epitope combinations. This analysis could provide useful information on whether the gut microbiome amplifies most tumor-specific T cells in a nonspecific manner or whether the amplification is restricted to a selected number of epitopes.

Fourth, to alter the composition of the gut microbiome we used the *Bifidobacterium* cocktail that did not colonize BALB/c but rather affected the abundancy of a few species (*Muribaculaceae* and *Lachnospiraceae* (Figure 4C)), which are the top priority candidates to be tested in as antitumorigenic/protumorigenic strains. However, the direct involvement of these species in tumor development awaits experimental evidence. It is relevant to point out that while the role of the microbiome in cancer immunity is now undisputable, uncertainties still exist regarding the identity of antitumor microbial species. In general, the sequencing of the gut microbiome has so far revealed that responders to checkpoint inhibitors have a more diverse population of bacteria, and the abundancy of some species appears to be associated with health (*Clostridiales, Ruminococci, Bifodobacterium, Fecalibacterium prausnitzii, Akkermansia muciniphila,* etc.) [4]. However, “beneficial” species vary from report to report, probably depending upon the models used or the tumor patients analyzed. Paradoxically, in studying the effect of the gut microbiome in a C57BL/6 mouse model of ADT therapy, Huribe-Herranz et al. [40] reported that mice heavily colonized with *Muribaculaceae* responded poorly to the antitumor therapy, a result which appears to be in contrast with what we have found in our study. Recently, a pool of eleven species have been identified from the human microbiome, which seems to broadly protect against different cancers/cancer models [41]. A retrospective analysis of the presence of some of the species included in the eleven-microbial pool might reconcile the discrepancies among the studies so far described.

The mechanisms through which the gut microbiome influences cancer immunity deserve a final comment. A number of not mutually exclusive mechanisms have been postulated [3,4]. First, the gut microbiome releases metabolites, such as polyamine, vitamin B16 and short-chain fatty acids (SCFAs), which mediate systemic effects on the host [42]. Second, live bacteria migrating from the intestinal mucosa have also been isolated at the tumor sites [43] where they can promote tumor inhibition through the activation of tumor-associated immune cells. Third, the microbiome could exert a long-distance effect by releasing products [37,44] and cytokines [3,6]. For instance, it has been shown that TLRs and NOD2 ligands mediate the effect of *Enterococcus hirae* and *Bacteroides fragilis* [37,43]. Interestingly, probiotics mediate proliferation of NK cells via DC maturation, and the stimulation of NK cells can both enhance their killing capacity toward tumor cells and induce tumor cell differentiation by IFN-γ release [45,46,47,48]. Fourth, the plethora of antigens expressed by the gut microbiome carry sequences homologous to cancer neo-epitopes (“molecular mimicry (MM)” hypothesis). Such homologous epitopes can activate resident DCs, which in turn induce T cell populations, cross-reacting with cancer neo-epitopes presented by tumor cells and/or by tumor-associated DCs. Usually residing in the *lamina propria*, these cross-reactive T cells (or activated DCs) can migrate to the tumor site and promote tumor-cell killing. For instance, it has been shown that *A. muciniphila* promotes the accumulation of CCR9^+^ T cells, and such cells are subsequently found among tumor-infiltrating T cells [7]. The MM hypothesis is particularly attractive since it would assign a previously unpredicted specificity to the antitumor activity of the gut microbiome. The experimental evidence supporting the existence of cross-reactive epitopes is still limited. It has been shown that the rare (<2%) long-term (>10 years) survivors of pancreatic cancer carry infiltrating cytotoxic T cells specific for a MUC16 neo-epitope which cross-reacts with pathogen-associated epitopes [49]. Moreover, bioinformatic analysis of the gut microbiome has revealed the existence of several microbiome antigens with high homology to known immunogenic T cell epitopes of bacterial, viral, and allergic antigens. This has led to propose the existence of microbiome “tolerogenic” and “inflammatory” epitopes which can dampen or increase the immunogenicity toward the homologous antigen-specific T cell epitopes [50,51]. Interestingly, allergic patients carry a less diversified microbiome with respect to healthy individuals and have a reduced number of “tolerogenic” epitopes, thus providing an explanation of the insurgency of the allergic disease. More recently, it has been elegantly proposed, using a mouse model of spontaneous autoimmune myocarditis, that mimic peptides from commensal bacteria can promote inflammatory cardiomyopathy in genetically susceptible individuals, leading to myocarditis and lethal heart disease [52]. Finally, it has been shown that mice bearing the tail-length tape measure protein (TMP) of a prophage found in the genome of the bacteriophage *Enterococcus hirae* mounted a TMP-specific CD8^+^ T-lymphocyte response upon immunotherapy with anti–PD-1 antibodies. Moreover, the administration of bacterial strains engineered to express TMP epitopes improved immunotherapy in mice [53].

**Figure 5:**
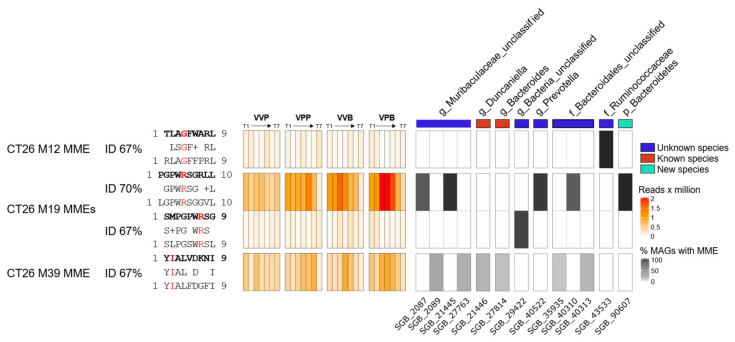
Heatmap representing abundances of 4 MMEs and bacterial species in which each MME is present. Each MME was selected for the presence of the CD8 CT26 tumor mutation, presence in at least 5 mice, with a predicted MHC I-binding percentile rank less than 0.5 and an identity higher than 60% against the CT26 CD8 neo-epitopes. On the left side are reported MME’s abundances expressed as reads per million. On the right side are reported the species in which that specific MME was found (black) inside the assembled metagenome. VPB: Pentatope OMVs + Bifidobacterium gavage; VPP: pentatope OMVs; VVB: empty OMVs + Bifidobacterium gavage; VVP: empty OMVs.

In a preliminary attempt to investigate whether the MM hypothesis could partially explain the results of this work, we performed an alignment of the amino acid sequences of four CD8+ T cells neo-epitopes described by Kreiter et al., 2015, against the metagenomic assemblies. Peptides with at least 60% homology with original neo-epitopes were filtered for the presence of the corresponding tumor mutation and prioritized on the basis of peptide/MHC I-binding affinity (see Materials and Methods for details). The ultimate goal was to see whether homologous peptides could be found in those microbial species which were mostly affected by the treatment (tumor challenge, gavages, vaccination, gavages and vaccination). We identified four high-affinity (“strong binders”) microbiome molecular mimicry epitopes (MMEs) that were present in the gut microbiome, contained one tumor mutation, and were homologous (>60% homology) to one of the CT26 neo-epitopes. In particular, Figure 5 shows the abundance of the four MMEs found in the gut microbiome and in which bacterial species those epitopes are present. Interestingly, the MME with the sequence LGPWRSGGVL that is 70% homologous to the M19 CD8+ T cell neo-epitope described by Kreiter et al. was found in some *Bacteroidales*, *Prevotella* and *Muribaculacee* species, and its abundance increased over time in treated mice, which showed a reduced tumor volume with respect to control animals. In particular, in these three taxa metagenomes, the LGPWRSGGVL MME peptide was found within the amino acid sequences of 15 highly conserved “hypothetical proteins”, with a sequence identity ranging from 56% to 100%. Considering that M19 was described by Kreiter at al. as being one of the most immunogenic CT26 neo-epitopes, it is tempting to speculate that M19-specific T cells elicited by the gut microbiome might contribute to the observed antitumor activity.

## 5. Conclusions

This work shows for the first time that microbiome composition can affect the efficacy of neo-epitope-based cancer vaccines, further extending the importance of intestinal bacteria in cancer immunity. From a translational standpoint, our work suggests that, to be most effective, personalized cancer vaccination should be combined with anticancer therapeutic modalities aiming at remodeling the intestinal microbiome. In support of this, we have shown that the growth of CT26 tumors in BALB/c mice was partially inhibited when oral gavages of *Bifidobacterium* were given to challenged mice, and that the combination of *Bifidobacterium* administration and vaccination resulted in a protection which was superior to vaccination alone and to *Bifidobacterium* administration alone. The beneficial effect of the combination also correlated with an increase in the frequency of vaccine-specific T cells. Importantly, the gut microbiome analysis by 16S rRNA gene sequencing and shotgun metagenomic sequencing showed that the abundancy of certain microbial species directly or inversely correlated with tumor progression, and that the oral administration of *Bifidobacterium* affected the relative abundance of such species, favoring the persistence of beneficial bacteria in the intestine. Interestingly, in the predicted proteome of *Bacteroidales*, *Prevotella* and *Muribaculacee* species, whose abundance directly correlates with a reduced tumor volume, we identified one peptide which shows strong homology to a highly immunogenic CT26 neo-epitope (M19). This observation might suggest that the microbiome can at least partially affect cancer immunity by eliciting T cells recognizing cancer neo-epitopes.

## Figures and Tables

**Table 1:** List of the synthetic peptides with the corresponding amino acid sequence.

Peptide Name	Aminoacid Sequence
M03	DKPLRRNNSYTSYIMAICGMPLDSFRA
M20	PLLPFYPPDEALEIGLELNSSALPPTE
M26	VILPQAPSGPSYATYLQPAQAQMLTPP
M27	EHIHRAGGLFVADAIQVGFGRIGKHFW
M68	VTSIPSVSNALNWKEFSFIQSTLGYVA

## Data Availability

Data are available upon request to the corresponding author.

## Supplementary Materials

The following are available online at www.mdpi.com/article/10.3390/vaccines9111356/s1, Figure S1: Frequency of Pentatope-specific CD4+ and CD8+ T cells, Figure S2: MDS analysis on all 16s rRNA samples colored by timepoints and conditions’ centroids., Figure S3: Differentially abundant species between T3 and T7., Figure S4: Differentially abundant species between T3 and T7.
