# Supplementary material for: Commensal Bifidobacterium Strains Enhance the Efficacy of Neo-Epitope Based Cancer Vaccines
Source: Vaccines (Basel). 2021 Nov 18;9(11):1356. doi: 10.3390/vaccines9111356 (PMC8619961; doi:10.3390/vaccines9111356)
Supplement: Supplementary file 1 [file vaccines-09-01356-s001.zip › vaccines-1433549-supplementary.pdf]

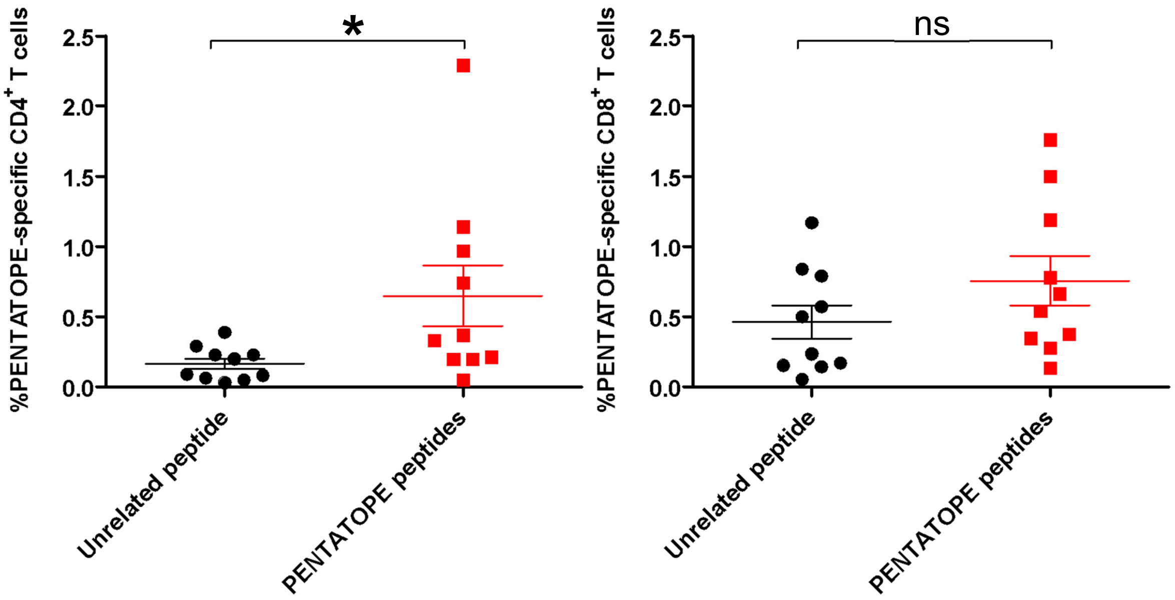

Figure S1: *Frequency of Pentatope-specific CD4⁺ and CD8⁺ T cells. Mice were vaccinated on day 0 and day 7 with 20 γg of Pentatope OMVs, a mixture (4 γg each) of 5 engineered OMVs, each one decorated with one of the five CT26 selected neoepitopes (20 γg in total). On day 12, spleens were collected from each mouse and the frequency of IFN-γ-producing, Pentatope-specific CD4⁺ and CD8⁺ T cells was determined by flow cytometry after splenocyte stimulation with Pentatope peptides or an unrelated peptide as a negative control (see Materials and Methods for details). Statistical analysis was performed using unpaired, one-tailed Student's t-test. *P < 0.05; ns, not significant.*

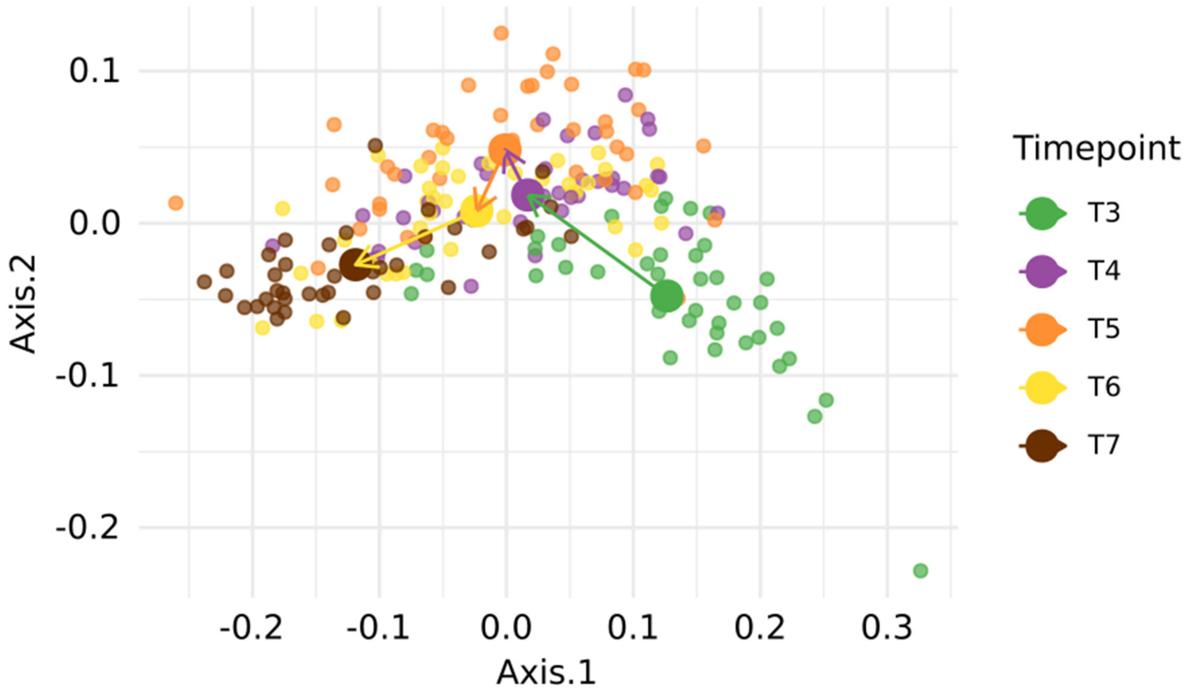

Figure S2: *MDS analysis on all 16s rRNA samples colored by timepoints and conditions' centroids. MDS analysis on all samples colored by timepoints and conditions' centroids (calculated by averaging intra-timepoint Weighted UniFrac distances) are highlighted. The analysis showed a continuous change in microbiome composition through timepoints. While the injection of the sole tumor induced a trajectory based on the decrease on the first MDS component and an increase on the second one, Bifidobacterium was able to alter the direction on the second component.*

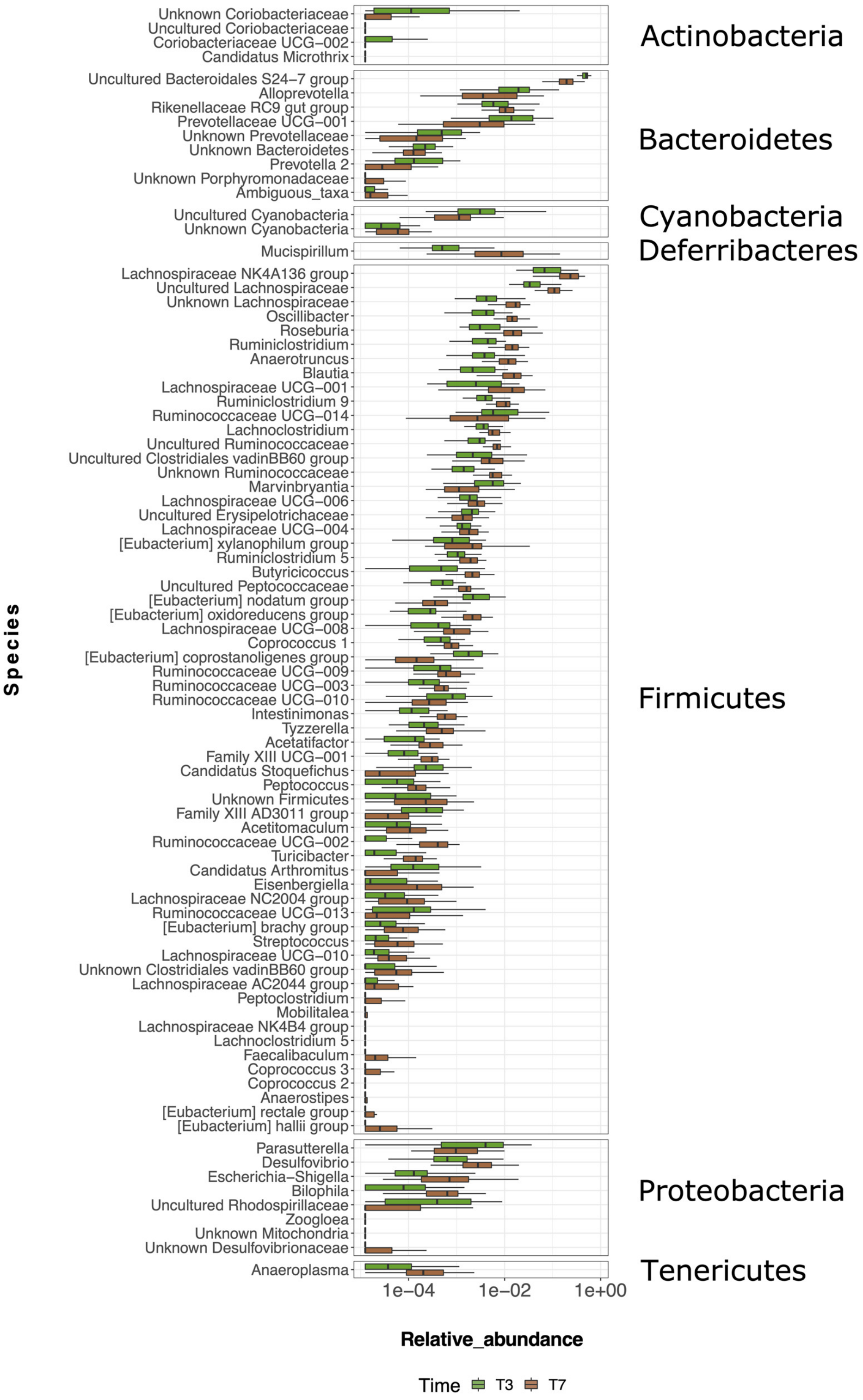

Figure S3: *Differentially abundant species between T3 and T7. The 89 differentially abundant species (Wilcoxon test) identified by 16s rRNA sequencing. Only species found to be significantly differentially abundant after false discovery rate error correction (p_{adj}<0.05) are reported.*

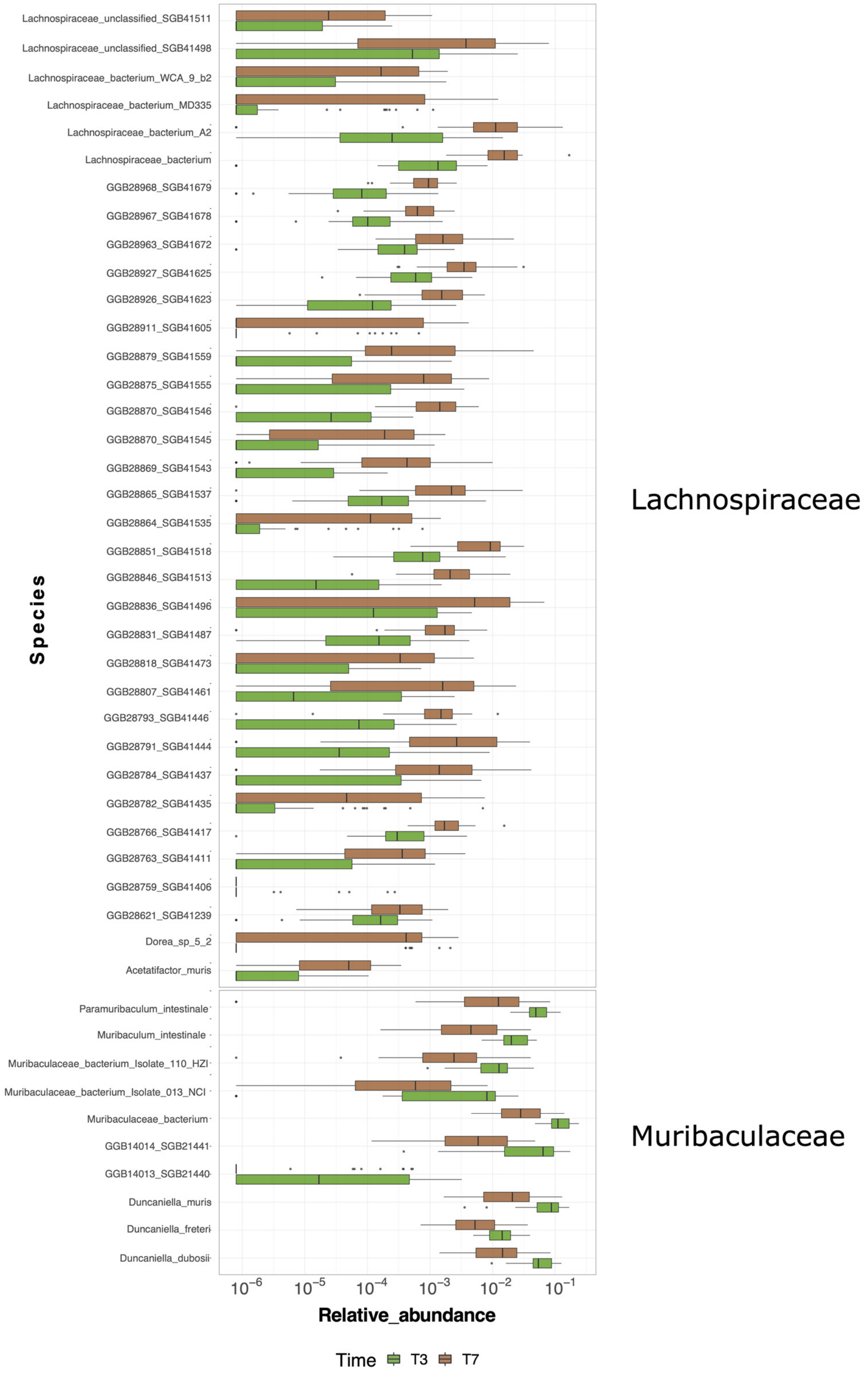

Figure S4: *Differentially abundant species between T3 and T7. The 45 differentially abundant species (Wilcoxon test) identified by shotgun metagenomics sequencing focusing on Muribaculaceae and Lachnospiraceae families. Only species found to be significantly differentially abundant after false discovery rate error correction (p_{adj}<0.05) are reported.*
